# Supplementary material for: Assembling covalent organic framework membranes via phase switching for ultrafast molecular transport
Source: Nat Commun. 2022 Jun 7;13:3169. doi: 10.1038/s41467-022-30647-3 (PMC9174484; doi:10.1038/s41467-022-30647-3)
Supplement: Supplementary file 1 — Supplementary Information [file 41467_2022_30647_MOESM1_ESM.pdf]

Supplementary Information

Assembling covalent organic framework membranes *via* phase switching for ultrafast molecular transport

Niaz Ali Khan[1,2], Runnan Zhang[1,2,3,4]*, Xiaoyao Wang [1,2], Li Cao[1], Chandra S. Azad[5], Chunyang Fan[1,2], Jinqiu Yuan[1,2], Mengying Long[1,2], Hong Wu[1,3,4,6]*, Mark. A. Olson[7] and Zhongyi Jiang[1,2,3,4]*

[1]Key Laboratory for Green Chemical Technology of Ministry of Education, School of Chemical Engineering and Technology, Tianjin University, Tianjin 300072, China.

[2]Collaborative Innovation Center of Chemical Science and Engineering (Tianjin), Tianjin 300072, China

[3]Haihe Laboratory of Sustainable Chemical Transformations, Tianjin 300192, China.

[4]Zhejiang Institute of Tianjin University, Ningbo, Zhejiang 315201, China.

[5]Department of Chemistry, Northwestern University, 2145 Sheridan Rd., Evanston, IL 60208 USA

[6]Tianjin Key Laboratory of Membrane Science and Desalination Technology, Tianjin University, Tianjin 300072, China.

[7]Department of Physical and Environmental Sciences, Texas A&M University Corpus Christi, 6300 Ocean Dr., Corpus Christi, TX 78412 USA

*E-mail: runnan.zhang@tju.edu.cn; *E-mail: wuhong@tju.edu.cn; *E-mail: zhyjiang@tju.edu.cn.

1. Materials and Methods

Reagents, Solvents and Materials

1,3,5-triformyl phloroglucinol (TFP), 1,3,5-triformylbenzene (TFB), 1,4-phenylenediamine (PDA) and methylene blue (MeB) were purchased from HEOWNS chemicals Co., Ltd. 4,4',4''-(1,3,5-triazine-2,4,6-triyl)trianiline (TTA), acetic acid, N, N-Dimethylacetamide (DMAC), o-dichlorobenzene (DCB) and n-butanol (BuOH) were purchased from Meryer Chemical Technology Co., Ltd. Alcian blue (AB), Congo red (CR) protoporphyrin IX, (PPh-IX), methyl blue (MB), and orange G (OG) were purchased from Shanghai Aladdin Bio-Chem Technology Co.,LTD. Indium tin oxide (ITO) coated glasses support were purchased from Luoyang Guluo Glass Co., Ltd. PTFE substrates were provided by Haining Chuangwei Filter Equipment Company Co. Ltd. All chemicals were used as received without further processing. Milli-Q water purification system was used to prepare Deionized water (DI water).

Methods

Phase switching strategy using two-steps procedure

Pre-assembly step in liquid phase toward pristine membranes

For the fabrication of ≈ 150 nm ultrathin membranes, TFP (2.10 mg, 0.01 mmol), 1,3,5-triformylbenzene (TFB, 1.62 mg, 0.01 mmol), PDA (1.62 mg, 0.015 mmol), TTA (3.54 mg, 0.01 mmol) were dissolved in separate vials in DMAc. DMAc was chosen in this step due to high solubility of amine and aldehyde monomers in DMAc which reduces reaction volumes (TFP≈ 23mg/ml, PDA≈25mg/ml and TTA≈17 mg/ml) and relatively high boiling point of DMAc, which wins sufficient evaporation time for the completion of the polymerization at this step. Next, the mixed solution containing TFP/PDA, TFP/TTA or TFB/PDA was poured on Indium Tin Oxide (ITO) coated disk; the solvents were evaporated at 60 °C to obtain pristine membranes. For the fabrication of thicker membranes, the concentration of monomers was increased keeping the same molar ratio.

Assembly step in vapor phase toward COF membranes

The pristine membranes from the pre-assembly step were placed on top of a glass bottle in a Teflon lined autoclave. Solvents (oDCB, BuOH) and catalyst (AcOH) in a volumetric ratio of 1:1:0.1

were poured on bottom of the Teflon vessel, at least 5 cm below the membrane surface. The whole assembly was heated at 145 °C for 18 h to obtain COF membranes. Free- standing COF membranes were obtained by etching ITO layer through dilute HCl.

Solvothermal strategy

The pristine membrane from the pre-assembly step was also treated in the liquid phase containing oDCB, BuOH and AcOH in a volumetric ratio of 1:1:0.1, and heated at 145 °C for 18 h. Free-standing COF membranes were obtained by etching ITO layer through dilute HCl.

Characterization

The Powder X-ray diffraction (PXRD) data were collected on a Rigaku D/max 2500v/pc equipment in the range of 3-35°. The membranes were etched and mounted on the XRD holder for characterization. The XRD simulations of all COF membranes were carried out using Reflex module of Materials Studio (MS) software. High resolution transmission electron microscopy (HTEM) (Tecnai G2 F20) was employed to obtain high resolution diffraction pattern of samples. The membranes were crushed in ethanol and drop casted on Ultrathin Carbon Film on Lacey Formvar/Carbon Films (Beijing Zhongjingkeyi Technology Co.,Ltd. BZ11032a) TEM grids. The Fourier transform infrared (FT-IR) spectra were recorded at a arrange from 500-4000 cm^{-1} on a BRUKER Vertex 70 spectrometer. Scanning Electron Microscopy (SEM) images were observed by field emission scanning electron microscope (Nanosem 430). N_2 adsorption/desorption isotherms were recorded on PS2-1055-B gas adsorption analyzers at 77 K using a liquid nitrogen bath. Several membranes were first fabricated and then grinded for porosity evaluation. All the membrane samples were degassed at 120 °C for 6 h under vacuum before N_2 analysis. For X-ray photoelectron spectroscopy (XPS) characterizations, the membranes were directly mounted on holders and characterized.

Performance evaluation

Molecular transport experiments

A lab made dead-end filtration cell (active area of 4.1 cm^2), equipped with magnetic stirrer and nitrogen flow for pressure maintenance, was used for molecular transport. DI water was used for water transport while reagent grade organic solvents were used for organic solvent nanofiltration

experiments. All dyes except (PPH-IX, dissolved in ethanol)) were dissolved in water for molecular separation. The concentration of dyes ranged from 50-1000 ppm. The permeance was stabilized by using an initial pressure of 3.5 bar. To make sure that the COF membranes do not act as adsorbent[1], the first 10 ml permeate of each test was discarded and then the concentration in the permeate was calculated at different intervals (10ml each for 20 cycles). Similarly, the permeance was also recorded at different pressures ranging from 0.5-3.5 bar. Long term water permeance was obtained by recording data at various intervals at 1 bar. Similarly, the performance was evaluated at various cycles of operation. After each cycle, the membrane was washed with water before next cycle.

2. Supplementary Figures

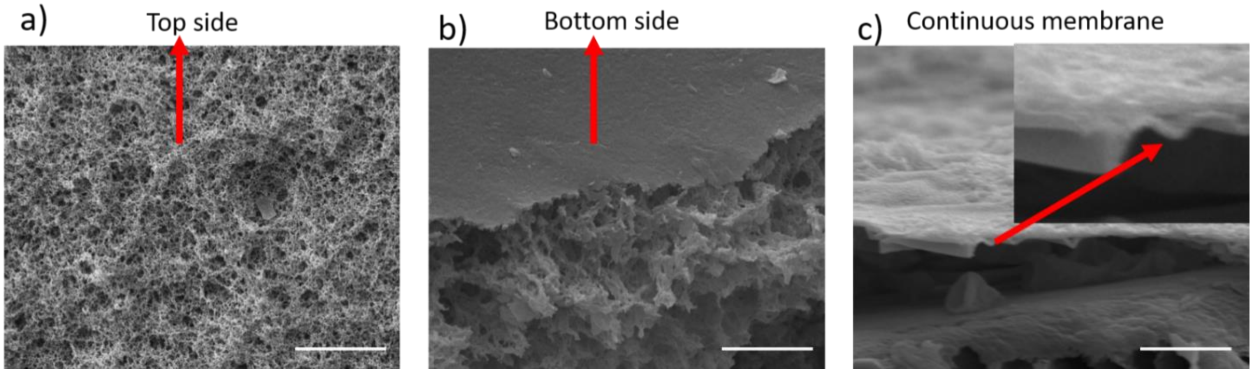

Supplementary Fig. 1. SEM image of thick membranes showing (a) the top and (b) bottom surface of COF membranes. (c) represents the ultra-thin membranes obtained after removing the top layer through adhesive tape. Scale bar, (a) 50 µm, (b, c) 1 µm.

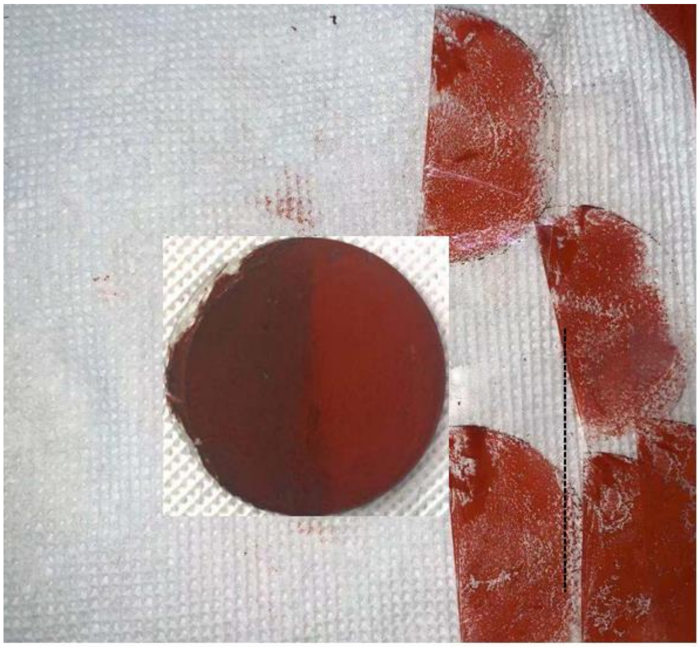

Supplementary Fig. 2. Digital photos of removing the top layer using adhesive tape.

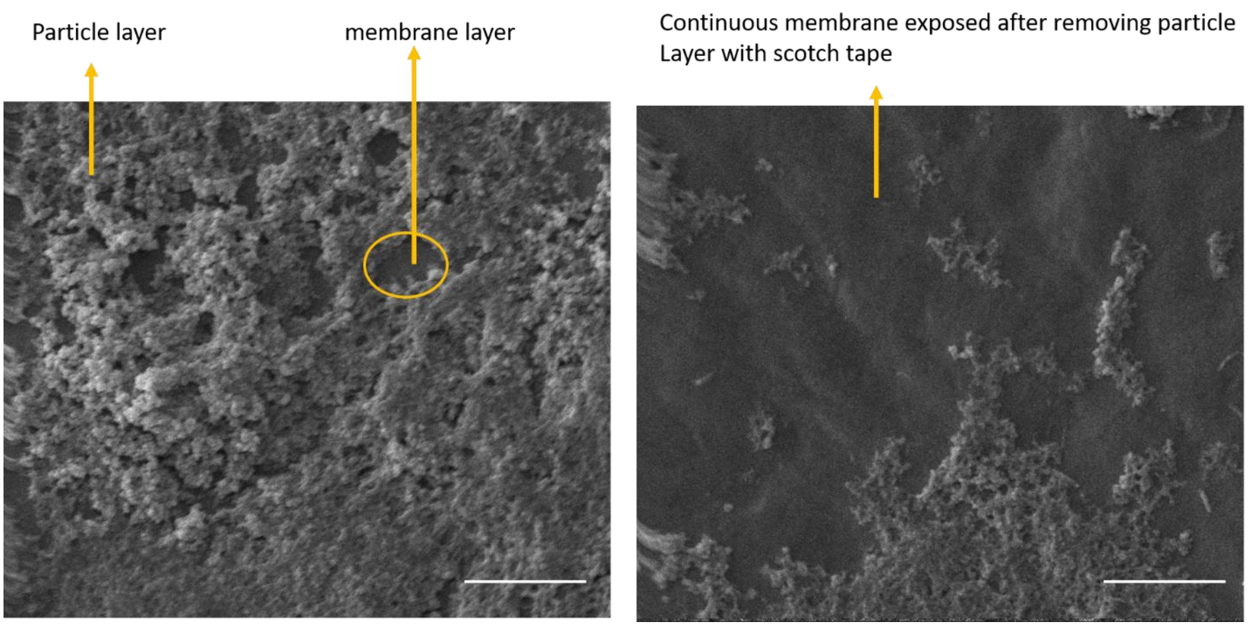

Supplementary Fig. 3. SEM of membranes top and bottom layer; before and after removing the top layer with adhesive tape. (Scale bar, 2 µm).

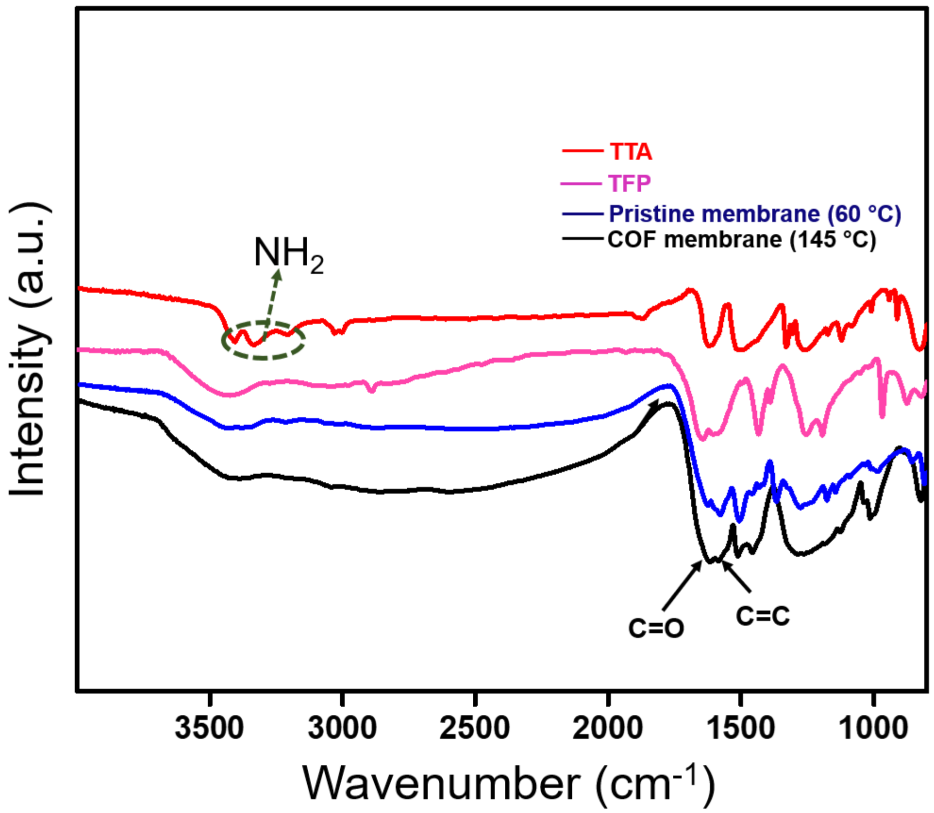

Supplementary Fig. 4. FT-IR of the TFP and TTA monomers, TFP-TTA pristine and COF membrane.

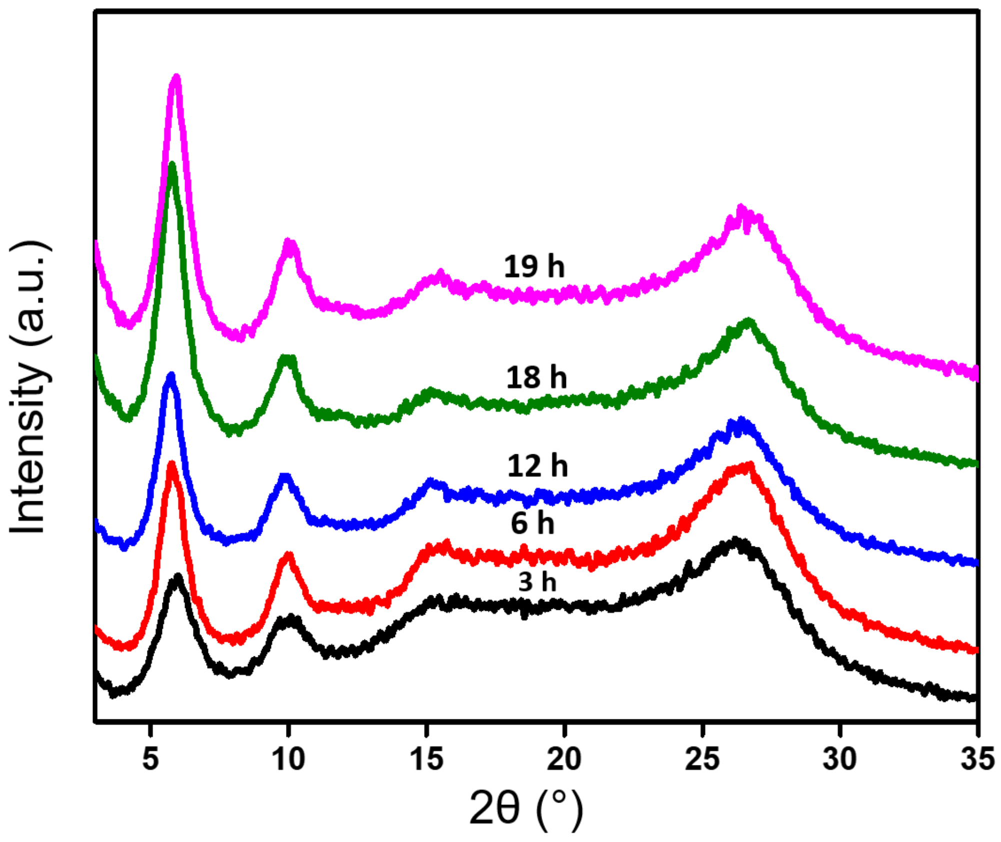

Supplementary Fig. 5. Time dependent XRD of TFP-TTA membranes at different time intervals. The intensity of peak at lower 2θ value increases with time and remains similar after 18 h.

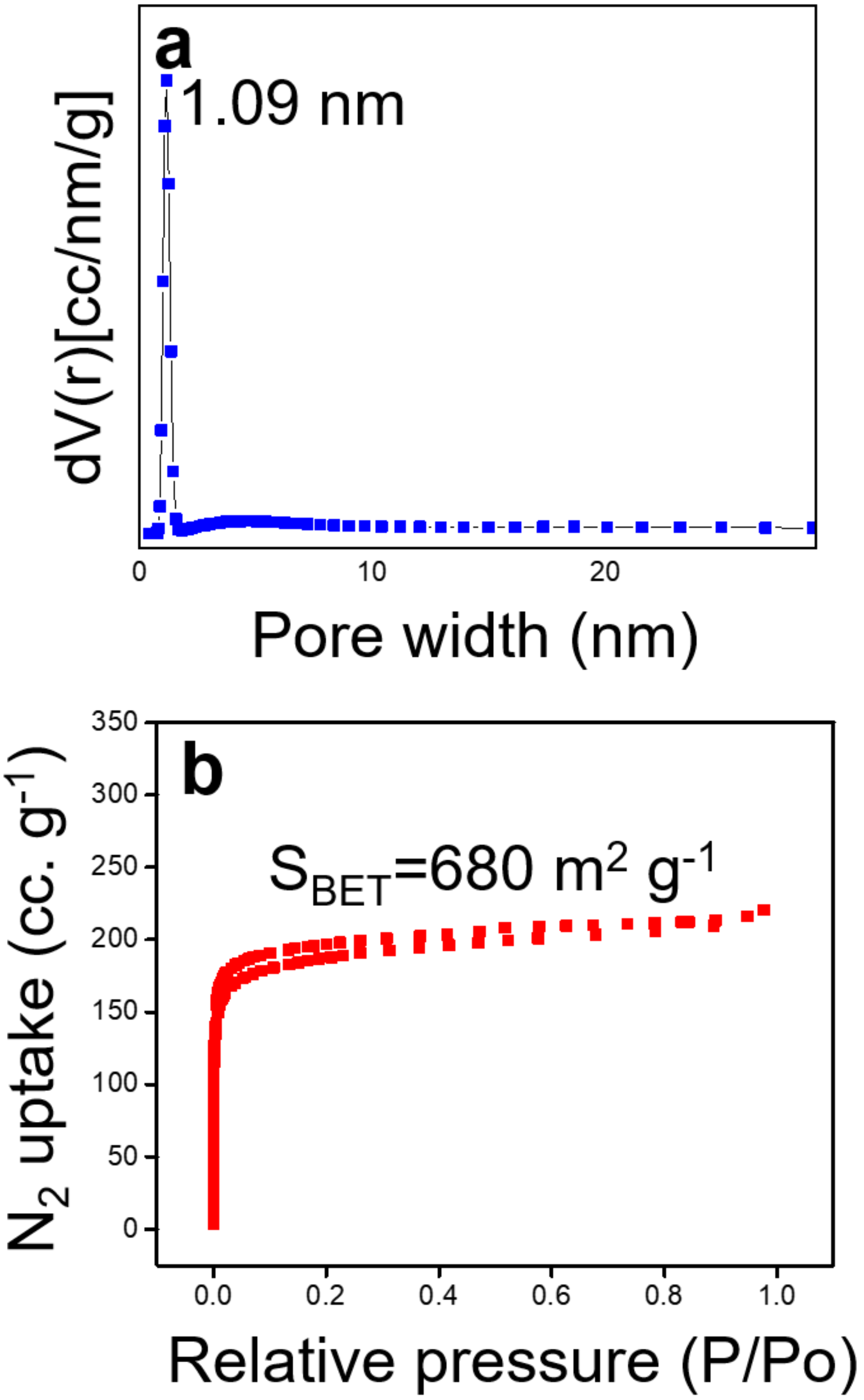

Supplementary Fig. 6. BET of TFP-TTA membrane showing pore size (a) and surface area (b).

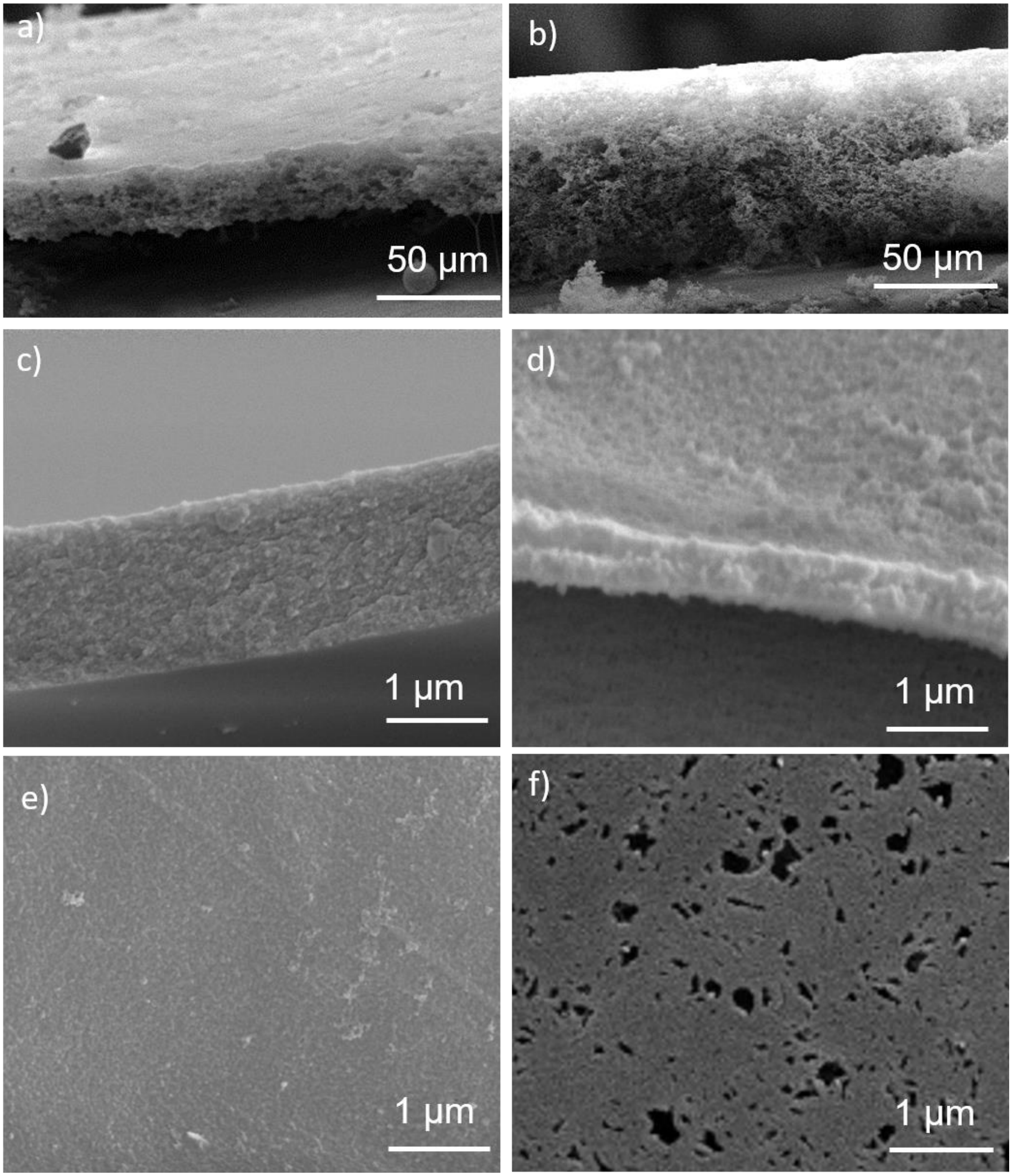

Supplementary Fig. 7. SEM of TFP-PDA membranes. a) phase-switching strategy, b) solvothermal strategy. Membranes after removing the top layer through adhesive tape, c) phase-switching strategy, d) solvothermal strategy. surface of membranes, e) phase-switching strategy, f) solvothermal strategy.

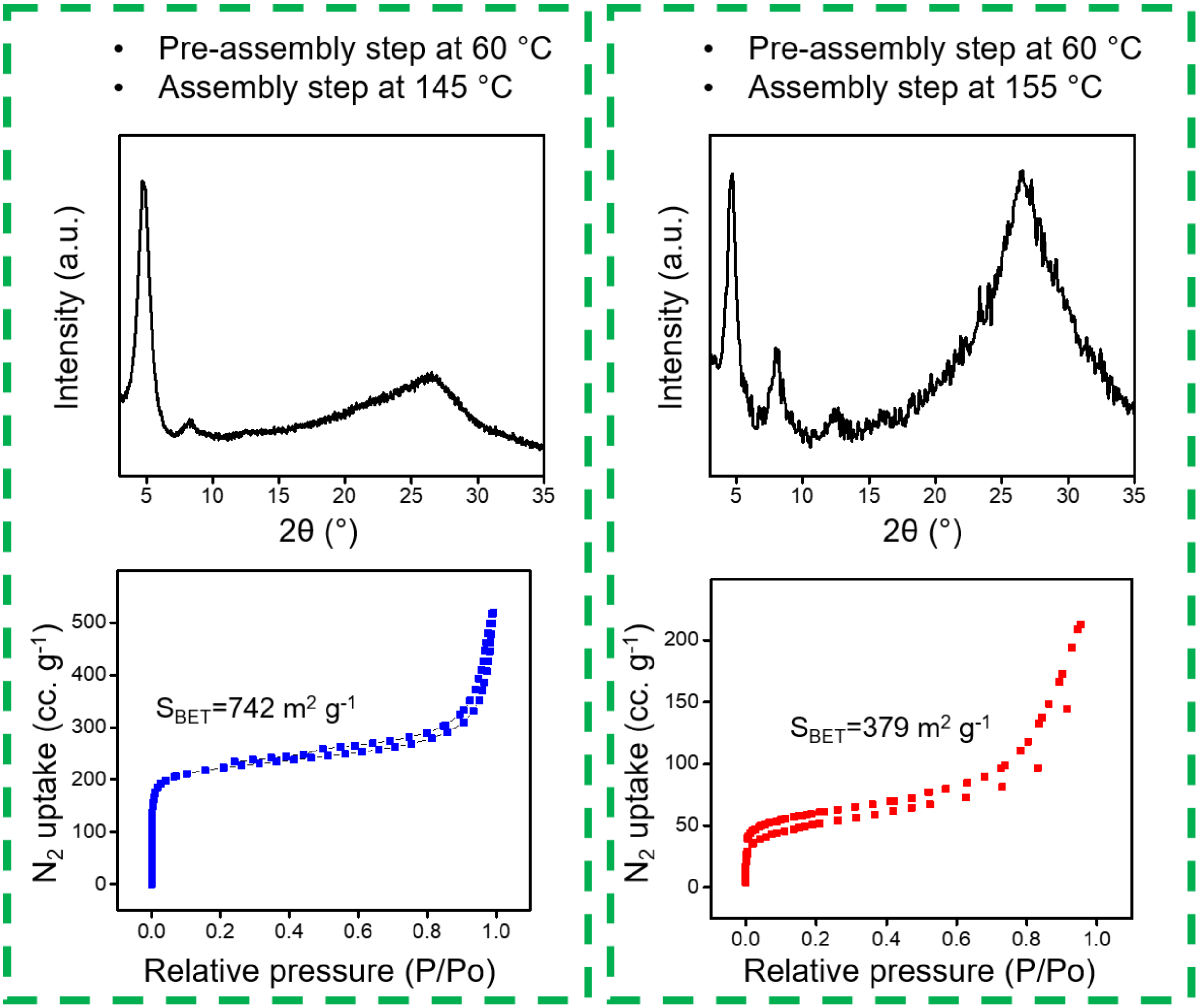

Supplementary Fig. 8. XRD and BET surface area of TFP-PDA membranes pre-assembled at 60 °C, (left) assembled at 145 °C, (right) assembled at 155 °C.

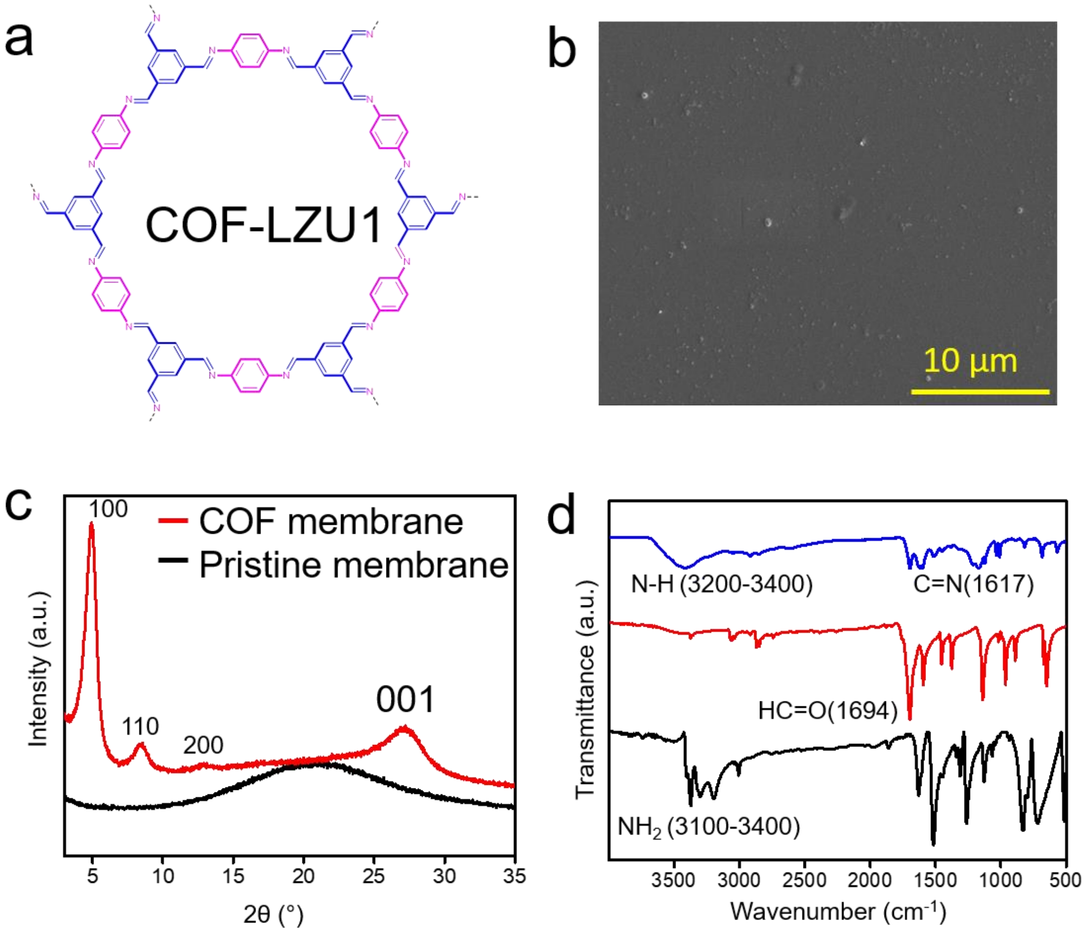

Supplementary Fig. 9. (a) COF-LZU1 chemical structure, (b) SEM exhibiting large defect-free area of the membrane, (c) XRD of the pristine and COF-LZU1 membranes, (d) FT-IR spectra of 1,4-diaminobenzene (black), 1,3,5-triformylbenzene (red), and COF-LZU1 membrane (blue).

3. Supplementary Tables

Supplementary Table 1. Molecular transport and separation evaluation of TFP-PDA membranes after specified time, prepared at various temperatures.

**Time (h)**	**125 °C**		**135 °C**		**145 °C**		**155 °C**	
	Permeance ($L{\cdot}m^{-2}{\cdot}h^{-1}{\cdot}bar^{-1}$)	CR rejection (%)	Permeance ($L{\cdot}m^{-2}{\cdot}h^{-1}{\cdot}bar^{-1}$)	CR rejection (%)	Permeance ($L{\cdot}m^{-2}{\cdot}h^{-1}{\cdot}bar^{-1}$)	CR rejection (%)	Permeance ($L{\cdot}m^{-2}{\cdot}h^{-1}{\cdot}bar^{-1}$)	CR rejection (%)
0	800 ±10	55±3	799±12	55±2	798±7	52±2	804±12	54±3
3	759±9	57±4	751±10	59±3	743±6	55±2	712±7	71±3
6	700±11	58±3	633±11	63±3	675±7	63±2	633±8	78±3
9	641±10	61±3	612±12	69±3	581±5	68±1	458±9	80±4
12	613±9	68±2	533±13	76±2	539±5	72±2	399±7	82±2
15	593±7	71±2	475±10	79±3	475±4	86±1	302±8	84±3
16	561±6	73±3	462±9	81±2	434±4	95±2	275±7	89±2
17	552±6	75±2	451±7	82±3	419±2	98±1	209±8	91±3
18	513±5	78±3	444±5	83±2	403±4	99±1	173±9	99±2
19	497±6	82±3	429±6	84±2	389±2	99±1	162±6	99±2
22	463±4	86±1	415±4	85±2	353±2	99±1	148±8	99±3

Supplementary Table 2. Molecular transport and separation evaluation of TFP-TTA membranes after specified time, prepared at various temperatures.

**Time (h)**	**125 °C**		**135 °C**		**145 °C**		**155 °C**	
	Permeance ($L{\cdot}m^{-2}{\cdot}h^{-1}{\cdot}bar^{-1}$)	CR rejection (%)	Permeance ($L{\cdot}m^{-2}{\cdot}h^{-1}{\cdot}bar^{-1}$)	CR rejection (%)	Permeance ($L{\cdot}m^{-2}{\cdot}h^{-1}{\cdot}bar^{-1}$)	CR rejection (%)	Permeance ($L{\cdot}m^{-2}{\cdot}h^{-1}{\cdot}bar^{-1}$)	CR rejection (%)
0	793 ±12	42±8	793±12	43±7	795±13	43±8	794±12	43±8

3	731±8	47±8	711±10	51±9	699±6	57±2	674±8	64±5
6	682±11	51±6	648±11	54±6	614±6	66±2	588±7	75±4
9	611±9	58±6	598±11	63±5	534±5	70±1	439±7	83±4
12	554±8	62±4	521±12	77±6	500±6	79±2	327±7	88±3
15	515±8	65±4	455±9	81±3	415±4	86±1	271±9	92±4
16	489±7	68±3	437±7	84±3	379±4	90±2	200±8	98±2
17	446±5	75±3	400±6	88±4	338±3	99±1	131±6	99±3
18	405±4	80±3	367±5	92±3	309±3	99±1	102±7	99±2
19	377±4	85±3	351±5	94±3	281±3	99±1	91±5	99±2
22	325±4	90±2	312±4	97±3	218±3	99±1	72±4	99±2

Supplementary Table 3. Molecular transport performance of reported membranes based on COFs.

COF system	Fabrication method	Permeance ($L \cdot m^{-2} \cdot h^{-1} \cdot bar^{-1}$)	Reference number
TFP-PDA	Phase-switching	Water=403<br>Acetonitrile=519	(This work)
TFP-PDA	Solid-vapor IP	Water=411<br>Acetonitrile=589	[2]
Tp-Bpy	Liquid-liquid IP	Water=211<br>Acetonitrile=339	[3]
M-TpTD	Solvothermal<br>(*In-situ*)	Water=189<br>Acetonitrile=278	[4]
COF-LZU1	Solvothermal<br>(*In-situ*)	Water=76	[5]
COFM	Solvothermal<br>(Exfoliation)	Water= 178<br>Acetonitrile=280	[6]
TFP-DHF	Liquid-air IP	Water= 90<br>Acetonitrile=130	[7]

EB-COF:Br	Exfoliation	Water=48	[8]
GO-CTN	Exfoliation	Water=226	[9]
COF/PSF	Liquid-Liquid IP	Water=50	[10]
$TpTG_{Cl}$	Exfoliation	Water=31	[11]

References

1. Fenton JL, Burke DW, Qian DW, de la Cruz MO, Dichtel WR. Polycrystalline covalent organic framework films act as adsorbents, not membranes. *J. Am. Chem. Soc.* **143**, 1466-1473 (2021).

2. Khan NA*, et al.* Solid–vapor interface engineered covalent organic framework membranes for molecular separation. *J. Am. Chem. Soc.* **142**, 13450-13458 (2020).

3. Dey K*, et al.* Selective molecular separation by interfacially crystallized covalent organic framework thin films. *J. Am. Chem. Soc.* **139**, 13083-13091 (2017).

4. Kandambeth S*, et al.* Selective molecular sieving in self-standing porous covalent-organic-framework membranes. *Adv. Mater.* **29**, 1603945 (2017).

5. Fan H, Gu J, Meng H, Knebel A, Caro J. High-flux membranes based on the covalent organic framework cof-lzu1 for selective dye separation by nanofiltration. *Angew. Chem. Int. Ed.* **57**, 4083-4087 (2018).

6. Halder A*, et al.* Ultrastable imine-based covalent organic frameworks for sulfuric acid recovery: an effect of interlayer hydrogen bonding. *Angew. Chem. Int. Ed.* **57**, 5797-5802 (2018).

7. Shinde DB*, et al.* Crystalline 2d covalent organic framework membranes for high-flux organic solvent nanofiltration. *J. Am. Chem. Soc.* **140**, 14342-14349 (2018).

8. Zhang WX, Zhang LM, Zhao HF, Li B, Ma HP. A two-dimensional cationic covalent organic framework membrane for selective molecular sieving. *J. Mater. Chem. A.* **6**, 13331-13339 (2018).

9. Khan NA*, et al.* Mixed nanosheet membranes assembled from chemically grafted graphene oxide and covalent organic frameworks for ultra-high water flux. *ACS Appl. Mater. Interfaces* **11**, 28978-28986 (2019).

10. Wang R, Shi XS, Xiao AK, Zhou W, Wang Y. Interfacial polymerization of covalent organic frameworks (COFs) on polymeric substrates for molecular separations. *J. Membr. Sci.* **566**, 197-204 (2018).

11. Zhang Z, Shi XS, Wang R, Xiao AK, Wang Y. Ultra-permeable polyamide membranes harvested by covalent organic framework nanofiber scaffolds: a two-in-one strategy. *Chem. Sci.* **10**, 9077-9083 (2019).
